# Machine learning based diagnostics of veterinary cancer on ultrasound and optical imaging data

**DOI:** 10.1080/01652176.2025.2510486

**Published:** 2025-05-30

**Authors:** Martynas Maciulevičius, Greta Rupšytė, Renaldas Raišutis, Mindaugas Tamošiūnas

**Affiliations:** a Research Institute of Natural and Technological Sciences, Vytautas Magnus University, Kaunas, Lithuania; b Department of System Analysis, Faculty of Informatics, Vytautas Magnus University, Kaunas, Lithuania; c Ultrasound Research Institute, Kaunas University of Technology, Kaunas, Lithuania; d Department of Electrical Power Systems, Faculty of Electrical and Electronics Engineering, Kaunas University of Technology, Kaunas, Lithuania; e Institute of Atomic Physics and Spectroscopy, University of Latvia, Rīga, Latvia

**Keywords:** Lipoma, soft tissue sarcoma, mast cell tumor, ultrasound imaging, fluorescence, white light, machine learning

## Abstract

Study advances current diagnostic efficiency of canine/feline (sub-)cutaneous tumors using machine learning and multimodal imaging data. White light (WL), fluorescence (FL) and ultrasound (US) imaging were combined into hybrid approaches to differentiate between malignant mastocytomas, soft tissue sarcomas and benign lipomas. Support Vector Machine and Ensemble classifiers were optimized *via* sequential feature selection. US radio-frequency signals were quantitatively analyzed to derive the colormaps of six US estimates, corresponding to spectral and temporal domains of the acoustic field. This resulted in the quantification of 72 morphological features for US; as well as 24 and 12 – for WL and FL data, respectively. Resulting classification efficiency for mastocytoma and sarcoma using US data was >75%; US+FL − 75–80%; US+WL − 85–90% and US+OPTICS − 90–95%. ∼100% classification efficiency was achieved for the differentiation between benign and malignant tumors even using single WL feature for Ensemble classifier. US features, resulting in inferior classification efficiency, were competitive to superior optical, as they were selected during optimization to be added to or replace optical counterparts. Additional tissue differentiation was performed on z-stacks of US colormaps, obtained using 3D arrays of US radio-frequency signals. This resulted in ∼70% differentiation efficiency for mastocytoma and sarcoma as well as >95% for benign and malignant tissues. The obtained additional metric of classification efficiency provides complementary diagnostic support, which for Support Vector Machine can be expressed as: 90.3 ± 1.9% (US+WL)×71.2 ± 0.6% (US_Depth Profile_). This hybrid criterion adds robustness to diagnostic model and may be very beneficial to characterize heterogeneous tissues.

## 1. Introduction

Canine mortality rate due to neoplasms is up to 20% and increases to 45% for animals older than 10 years. Around 30% of all neoplasms appear on the skin. The most common skin and subcutaneous tumors are mast cell tumors (MCTs) (16.8–21%), lipomas (LPs) (8.5%), histiocytomas (8.4%) and perianal gland adenomas (7.8%) (Hauck 2013).

LPs are benign tumors, which, in general, are noninvasive and non-metastatic. Depending on the origination site, large LPs may create discomfort, occasionally leading to functional impairment of the animal (Kolb et al. 2024). Conversely to LPs, which can be well-differentiated cytologically, MCTs manifest in largerly heterogeneous morphological structure, ranging from benign to highly aggressive pathogenesis (Garrett 2014; de Nardi et al. 2022). Soft tissue sarcomas (STSs) are locally invasive malignancies, characterized by pronounced metastatic potential. Surgical excision is the primary therapeutic option, therefore, a defined surgical margin is necessary to diminish the risk of cancer recurrence (Hohenhaus et al. 2016; De Nardi et al. 2023).

In human dermatology skin tumors can be assessed by dermatoscopy, however, in veterinary a minimally invasive diagnostic method, called fine-needle aspiration cytology (FNAC), is performed (Wang et al. 2014). A veterinarian takes a biopsy from the tumor to prepare cytological samples for subsequent analysis using an optical microscope. Another method, histopathology, allows to evaluate a plethora of morphological features at a cellular and tissue levels (Avallone et al. 2021). Therefore, it remains a gold standard for cancer diagnostics. However, the interpretation of both FNAC and histopathology are strongly dependent on the experience of the pathologist. Also, diagnostic efficiency is largerly influenced by tumor heterogeneity, especially, for highly variable tumors, like MCT and STS. In this context, the development of biophysical imaging techniques, including ultrasound (US) and optical (white light, WL, and fluorescence, FL) may provide a valuable supplementary criteria, complementing conventional cytology or histopathology. These methods are not intended to replace gold-standard techniques of cytology or histopathology, but offer additional quantitative information, that can reduce diagnostic uncertainty for ambiguous cases. Indeed, a variety of physical modes, such as acoustic imaging (conventional or contrast-enhanced US) (Ohlerth and O’Brien 2007) or optical imaging (optical coherence tomography, OCT, or Raman spectroscopy) (Lages and Selmic 2021; Tamošiūnas et al. 2022) have already been studied in veterinary oncology. However, such methods as OCT or Raman spectroscopy are expensive and technically complex to be effectively incorporated into conventional veterinary settings. Thus, we propose a concept on multimodal diagnostics, integrating US, WL and FL imaging techniques, which in tandem with machine learning (ML) could provide diagnostic support to conventional histopathology.

Conventional imaging method is WL imaging, performed on (hematoxylin and eosin) H&E-stained histological samples, which provides essential information about the morphological characteristics of the tissue. It is widely used due to simple sample preparation, high resolution and accessibility. However, WL imaging is not sufficient to provide information on the internal structure of the tissue. On the contrary, the techniques of US imaging provide information on the tissue internal architecture. Sample exposure to US results in different pattern of back-scattered US waves, which effectively differentiate various tissue densities and compositions, based on the variability in acoustic impedance (Andrekute et al. 2016). Optical FL imaging is beneficial as it highlights the constituents of extracellular matrix, subcellular structures, indicates cellular density as well as, in particular cases, provide assistance in defining tumor margins (van Dam et al. 2011; Stibbe et al. 2023).

Our study focuses on multimodal imaging of LP, MCT and STS tissues, by employing the techniques of optical (WL and FL) imaging as well as US scanning acoustic microscopy (SAM). SAM, also termed, acoustic microimaging, is a nondestructive method, designed to detect defects in biological tissue or opaque solids. It allows for a detailed three-dimensional (3D) imaging of the internal architecture of the biological tissues (Miura et al. 2013; Akhtar et al. 2016). By scanning the surface of the sample, reflected US waves are registered. Subsequently, 3D image stacks are created from the obtained data using the algorithms of signal processing.

In histology, hematoxylin binds to nucleic acids and stains cell nuclei in blue or purple color, while eosin binds to cytoplasmic proteins, which appear pink or red under WL imaging. Autofluorescence efficiently highlights the components of extracellular matrix, mainly, elastin and collagen fibers (Fischer et al. 2008). Eosin also emits yellow to orange-red (580–600 nm) FL, when excited by 490–520 nm light (Lahiani et al. 2018). Recently, Wu et al. (2023) combined WL and autofluorescence imaging to examine the composition of stromal collagen, present in breast cancer tissue. The subsequent training of convolutional neural network (CNN) resulted in high classification (89.01%) and decision (87.53%) accuracy. Previously, Gesperger et al. (2020) used optical coherence microscopy, combined with FL imaging of 5-aminolevulinic acid (5-ALA), for the identification of surgical margins prior to brain cancer surgery. The application of CNN resulted in 97% accuracy and 100% specificity for the differentiation between cancerous and healthy tissues, leading to an increment in tumor margin definition.

Our dependency on the datasets, containing small amount of information, such as the characteristics of a single tumor or individual histological analysis, generally kept the amount of variables, rather, small. Therefore, standard statistical methods or even a veterinarian’s intuition could be used to derive a diagnosis. Optical or US images, obtained at high resolution, can reveal millions of subtle cellular features and produce dozens or hundreds of diagnostic parameters. In these situations, physician’s intuition and standard statistical methods do not generally apply (Cruz and Wishart 2007; Gurcan et al. 2013; Rabe et al. 2019). As a result, artificial intelligence (AI)-based techniques can discover and identify patterns and relationships using complex datasets, ensuring more accurate diagnosis. Recent research proves that AI-based diagnosis can be produced at the comparable level to human-derived in different fields. The diagnostic efficiency of deep learning algorithms, employed on histological samples, was comparable with the diagnostic metrics, provided by experienced pathologists in grading prostate (Nir et al. 2019: Ström et al. 2020; Ryu et al. 2019), breast (Ehteshami Bejnordi et al. 2018), lymphoma (El Achi and Khoury 2020) and lung cancer (Hägele et al. 2020). In comparison to conventional statistical methods, ML was evaluated to be more effective (up to 25%) in providing diagnosis of different types of cancer (Cruz and Wishart 2007). Therefore, some of the ML models have already been FDA-approved and adopted into clinical settings for the prediction of malignancy in pulmonary nodules (in CT-scans) (Massion et al. 2020) as well as prostate or breast cancer using digital histopathology (Kanan et al. 2020; da Silva et al. 2021). Furthermore, it has been demonstrated that the efficiency of AI-based cancer detection is enhanced, if different-nature physical diagnostic techniques are integrated. For instance, optical coherence tomography (OCT) or diffuse reflectance spectroscopy were applied in tandem with US imaging, Raman spectral band imaging or conventional dermatoscopy (Haenssle et al. 2018; Schneider et al. 2019; Tiwari et al. 2020; Tamošiūnas et al. 2022). However, the approach of multimodal imaging, integrating US, WL and FL, has not been investigated in veterinary oncology yet.

Aim of the study – to develop a comprehensive diagnostic framework, which integrates US, WL and FL imaging, to differentiate between malignant MCT and STS as well as benign LP tissues in canine and feline patients. Current study employs ML classifiers, conventional Support Vector Machine (SVM) as well as more advanced Random Forest, which is a type of Ensemble (ENS) learning. SVM is highly-recognized for its: i) efficiency in handling high-dimensional datasets, ii) capacity to create optimal decision boundaries, resulting in enhanced separation between classes (Li et al. 2018). The latter properties make SVM indispensable for the analysis of complex datasets, which require subtle distinctions, such as the differentiation between heterogeneous MCT and STS, based on the data of histological imaging. Meanwhile, ENS learning relies on the operation of multiple decision trees (DTs), applied for complex classification tasks, simultaneously reducing the risk of overfitting (Iranzad and Liu 2024). Current property is essential, when dealing with diverse tissue classes, like MCT and STS, often characterized by overlapping features.

Our approach aims to provide real-time diagnostic support for veterinary oncologists, who perform conventional cancer diagnostics using histopathological samples. MCT and STS have different pathogenesis – MCTs are occasionally malignant, and, therefore, require different surgical approaches and systemic treatments. Therefore, essential focus is given on the differentiation between MCT and STS tissues due to similar structural features and pronounced heterogeneity. Such distinctions are essential in providing optimal treatment protocols for veterinary patients. Current research is carried out using histologically confirmed diagnoses to differentiate pre-labeled tumor types. This is a proof-of-concept study, that cannot be used as an alternative to clinical validation for unknown cancer cases. The goal of our study is to create ML-based assistant, that could work alongside histopathology or FNAC, to support diagnostic decision for ambiguous cases. We envision our method as a supplementary aid to the conventional diagnostic techniques, rather than a stand-alone substitute.

## 2. Materials and methods

### 2.1. Tumor samples

The experiments were conducted on veterinary *ex vivo* clinical samples. The consent to participate was obtained from all animal owners. A total of 66 tumor samples were obtained from 51 animals: 18 LPs from 11 dogs and 2 cats, 30 STSs from 12 dogs and 11 cats, as well as 18 MCTs from 12 dogs and 3 cats. The average age of the animals was 8.3 years, ranging from 2 to 13 years. After surgical resection, the tumors were maintained in 10% formalin and subjected to histopathologic analysis. The initial processing involved tissue dissection to identify suitable examination areas, embedding in paraffin blocks and H&E staining. Additional details about the animal cohort and details, regarding tumors, are given in Appendix Table A1 in Supplementary material.

**Table 1.** Multimodal increment for the classification of MCT and STS tissues.

SVM								
	Metric							
Modes	Accuracy	Specificity	Sensitivity	NPV	Precision	F1 Score (−)	F1 Score (+)	Absolute Multimodal Increment
**WL** **→** **US+WL**	**86.1 **±** **0.2% **→** **90.3 **±** **1.8% [4.2 ± 2.5%]	**86.1 **±2.5% **→** **90 **±2% [3.9 ± 3.3%]	**86.1 **±3.2% **→** **90.6 **±2.8% [4.5 ± 2.9%]	**86.7 **±2.9% **→** **90.9 **±2.3% [4.2 ± 2.7%]	**86.5 **±2.2% → **90.2 **±1.8% [3.8 ± 2.9%]	**86.2 **±2.2% → **90.3 **±1.8% [4.1 ± 2.6]	**86 **±2.2% → **90.2 **±2% [4.2 ± 2.5]	**33/70**
**OPT** **→** **US+OPT**	**90 **±** **1.7% → **91.9 **±** **1.3% [1.9 ± 1.9%]	**91.1 **±2.2% → **91.1 **±2.1% [0 ± 3.6%]	**88.9 **±1.7% → **92.8 **±2.4% [3.9 ± 2.9%]	**89.2 **±1.6% → **93.2 **±2% [4 ± 2.4%]	**91.2 **±2.2% → **91.7 **±1.8% [0.5 ± 3.2%]	**90.1 **±1.7% → **91.9 **±1.3% [1.8 ± 2%]	**89.9 **±1.7% → **92 **±1.3% [2.1 ± 1.8%]	**44/70**
**US** **→** **US+FL**	**75 **±** **1.9% → **78.3 **±** **3% [3.3 ± 3.4%]	**75.6 **±2.8% → **78.9 **±4% [3.3 ± 4.6%]	**74.4 **±2.5% → **77.8 **±2.6% [3.4 ± 3.2%]	**74.9 **±2% → **77.9 **±2.6% [3 ± 3.1%]	**75.6 **±2.2% → **79.4 **±3.7% [3.8 ± 4.2%]	**75 **±2.1% → **78.2 **±3.2% [3.2 ± 3.6%]	**74.8 **±2% → **78.4 **±2.9% [3.6 ± 3.3%]	**54/70**
**WL** **→** **OPT**	**86.1 **±** **0.2% → **90 **±** **1.7% [3.9 ± 2%]	**86.1 **±2.5% → **91.1 **±2.2% [5 ± 3.2%]	**86.1 **±3.2% → **88.9 **±1.7% [2.8 ± 3.2%]	**86.7 **±2.9% → **89.2 **±1.6% [2.5 ± 2.8%]	**86.5 **±2.2% → **91.2 **±2.2% [4.7 ± 2.8%]	**86.2 **±2.2% → **90.1 **±1.7% [3.9 ± 2.1%]	**86 **±2.2% → **89.9 **±1.7% [3.9 ± 1.9%]	**40/70**
**WL** **→** **US+OPT**	**86.1 **±** **0.2% → **91.9 **±** **1.3% [5.8 ± 2.1%] **(*p* < 0.05)**	**86.1 **±2.5% → **91.1 **±2.1% [5 ± 3%]	**86.1 **±3.2% → **92.8 **±2.4% [6.7 ± 2.9%]	**86.7 **±2.9% → **93.2 **±2% [6.5 ± 2.7%]	**86.5 **±2.2% → **91.7 **±1.8% [5.2 ± 2.9%]	**86.2 **±2.2% → **91.9 **±1.3% [5.7 ± 2.3%] **(*p* < 0.05)**	**86 **±2.2% → **92 **±1.3% [6 ± 2.1%] **(*p* < 0.05)**	**46/70**
**ENS**								
	**Metric**							
**Modes**	**Accuracy**	**Specificity**	**Sensitivity**	**NPV**	**Precision**	**F1 Score (−)**	**F1 Score (+)**	**Absolute Multimodal Increment**
**WL** **→** **US+WL**	**83.9 **±** **2.5% → **86.9 **±** **1.9% [3 ± 2.4%]	**84.4 **±3.6% → **90 **±2.8% [5.6 ± 4.6%]	**83.3 **±3.1% → **83.9 **±3.7% [0.6 ± 2.8%]	**84 **±2.8% → **85.8 **±2.8% [1.8 ± 2.2%]	**85.1 **±3.2% → **90.2 **±2.5% [5.1 ± 4.2%]	**83.8 **±2.6% → **87.4 **±1.8% [3.6 ± 2.6]	**83.8 **±2.4% → **86.3 **±2.2% [2.5 ± 2.4]	**43/70**
**OPT** **→** **US+OPT**	**87.8 **±** **1.8% → **87.5 **±** **1.7% [−0.3 ± 2.4%]	**90 **±3.3% → **88.3 **±2.7% [−1.7 ± 3.6%]	**85.6 **±2.5% → **86.7 **±2.4% [1.1 ± 2.2%]	**86.6 **±2.1% → **87.3 **±2.2% [0.7 ± 2.1%]	**90.5 **±2.8% → **88.7 **±2.3% [−1.8 ± 3.3%]	**87.9 **±2% → **87.5 **±1.7% [−0.4 ± 2.5%]	**87.5 **±1.8% → **87.4 **±1.7% [−0.1 ± 2.2%]	**34/70**
**US** **→** **US+FL**	**73.6 **±** **1.7% → **76.7 **±** **2.6% [3.1 ± 2.7%]	**76.7 **±3.2% → **78.9 **±2.5% [2.2 ± 4%]	**70.6 **±2.6% → **74.4 **±4.8% [3.8 ± 5.1%]	**72.4 **±1.7% → **76.9 **±3.4% [4.5 ± 3.3%]	**75.9 **±2.4% → **77.8 **±2.3% [1.9 ± 3.1%]	**74.2 **±2% → **77.4 **±2.2% [3.2 ± 2.6%]	**72.7 **±1.8% → **75.5 **±3.3% [2.8 ± 3.4%]	**42/70**
**WL** **→** **OPT**	**83.9 **±** **2.5% → **87.8 **±** **1.8% [3.9 ± 1.6%]	**84.4 **±3.6% → **90 **±3.3% [5.6 ± 3%]	**83.3 **±3.1% → **85.6 **±2.5% [2.3 ± 1.9%]	**84 **±2.8% → **86.6 **±2.1% [2.6 ± 1.7%]	**85.1 **±3.2% → **90.5 **±2.8% [5.4 ± 2.4%]	**83.8 **±2.6 → **87.9 **±2% [4.1 ± 1.8%]	**83.8 **±2.4% → **87.5 **±1.8% [3.7 ± 1.4%]	**45/70**
**WL** **→** **US+OPT**	**83.9 **±** **2.5% → **87.5 **±** **1.7% [3.6 ± 2.3%]	**84.4 **±3.6% → **88.3 **±2.7% [3.9 ± 3%]	**83.3 **±3.1% → **86.7 **±2.4% [3.4 ± 3.3%]	**84 **±2.8% → **87.3 **±2.2% [3.3 ± 3.1%]	**85.1 **±3.2% → **88.7 **±2.3% [3.6 ± 2.5%]	**83.8 **±2.6 → **87.5 **±1.7% [3.7 ± 2.4%]	**83.8 **±2.4% → **87.4 **±1.7% [3.6 ± 2.4%]	**46/70**

Arrow indicates the change in classification metric, when increasing the number of imaging modes. The multimodal increment in absolute value and associated uncertainty (SEM) is given in square brackets. The proportion in column ‘absolute multimodal increment’ indicates the number of increased (in absolute value) classification metrics, summarized from all folds of outer partition (7 metrics/fold × 10 folds = 70 metrics). Green color highlights the groups with absolute increment in more than ½ of total metrics. Yellow color highlights statistically significant multimodal increment, obtained for particular metric after statistical evaluation using paired wilcoxon signed rank test. All other multimodal increments were evaluated to be statistically insignificant. Abbreviation ‘OPT’ refers to OPTICS.

### 2.2. Study design

**Figure 1.**
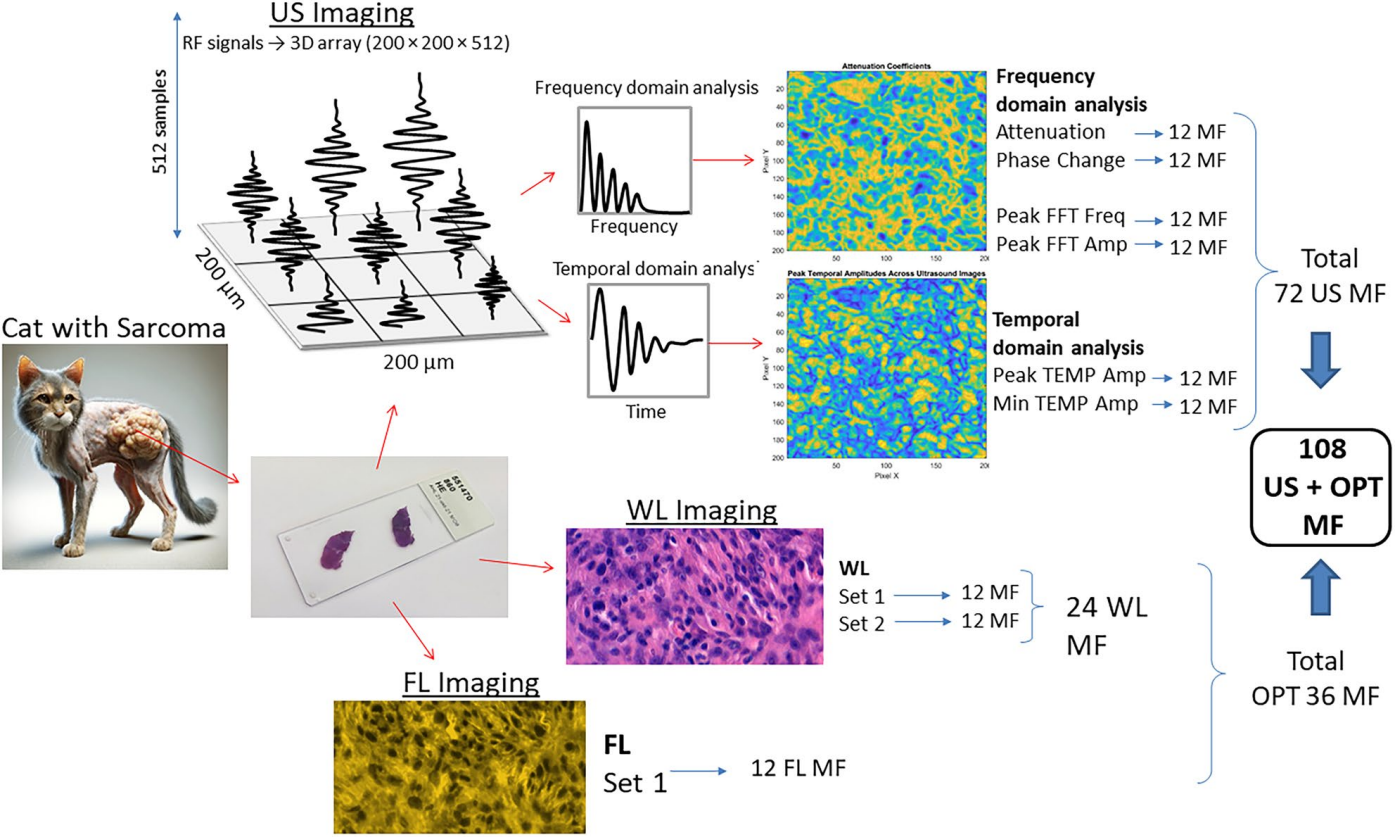
Study design, represented in successive steps. Tissue imaging, using US, WL and FL techniques, was followed by the quantification of computationally-defined equivalents of morphological features (MF) for US and optical (OPT) images. Eventually ML-based tissue classification was performed.

The schematic illustration of the study is presented in Figure 1. The histological samples were subjected to US and optical (WL and FL) imaging to reveal different features of the tissue. Subsequently, US radio-frequency (RF) signals were analyzed to produce the following colormaps (images) of US estimates, corresponding to fast Fourier transform (FFT) and temporal domains of the acquired RF signals. The obtained sets of images were used for the quantification of 12 morphological features for each US colormap. Overall, it produced 72 features for US, 24 for WL and 12 for FL data. Subsequently, the optimal set of features was determined during classifier training *via* sequential feature addition.

### 2.3. Imaging modalities

#### 2.3.1. Optical (WL and FL) imaging

Digitalization and imaging of H&E-stained slides was executed with a Pannoramic 250 FLASH III DX brightfield slide reader (3DHistech Kft., Budapest, Hungary) provided with a 20×/0.8 NA Plan-Apochromat (Carl Zeiss, Germany) objective. WL images were examined by an experienced veterinary histopathologist to identify the type of cancer and characterize the structural features of the samples. Autofluorescence images were recorded using a yellow fluorescence channel (YFL) in which endogenous fluorophores were excited by a 531/40 nm light source. Emitted fluorescence was detected using a 590/40 nm filter with an image resolution of 0.121 µm per pixel.

#### 2.3.2. US imaging

US microscope easySAM (center frequency of the transducer ∼350 MHz) (Kibero, Germany) was used to perform C-scans of histological slides (1 µm scanning step). The coverglass of the histological sample was removed before scanning and the void between the tissue and US transducer was filled with water. Produced images were of 200 × 200 µm size and 10 µm resolution. RF signals were acquired for each pixel, using a summation algorithm, resulting in 3D data array (200 × 200 × 512) for each US image. 200 × 200 represents the width and length dimensions (axes of scanning), while 512 represents the total number of samples within RF signal, corresponding to vertical (depth) or z-axis. Sampling rate was 2 × 10^9^ samples/s. For example, 1 × 1 × 512 corresponds to a single RF, composed of 512 samples, obtained for the first pixel (at spatial coordinates: *x* = 1, *y* = 1) within the histological sample.

### 2.4. Data analysis

#### 2.4.1. Optical (WL and FL) data analysis

Because of relatively small study cohort, the dataset, used in the current study, was arranged using the strategy of patch-level splitting. The regions of interest (ROIs) of 1500 × 800 pixels, corresponding to multiple locations of cancerous tissue, were manually selected from original images. A total of 10 ROIs were selected from each of 18 LP cases, 10 ROIs – from each of 18 MCT cases and 6 ROIs – from each of 30 STS cases. This data arrangement resulted in 180 image patches for each tumor class: LP, MCT and STS. The procedure was repeated for WL twice (the same tumor) in order to obtain two datasets WL1 and WL2, which were pooled into a single WL group. This was performed to: i) account for the heterogeneity of malignant tissues and ii) produce a larger pool of optical features, that would be competitive to US. For FL images a single set of 180 tissue patches (for each: LP, MCT and STS), was created. Each image patch was saved separately in Tag Image File Format (TIFF).

#### 2.4.2. US data analysis

10 US scans were performed for each LP or MCT case and 6 US scans – for each STS case. This resulted in a total of 180 US scans for each tissue class of LP, MCT and STS. US C-scans produced 180 US images, represented as three-dimensional arrays of RF signals. FFT was applied on RF signals, and the following US estimates were quantified to derive each pixel within the 200 × 200 matrix. This resulted in the colormaps of: **i) Attenuation, ii) Phase Change, iii)** Frequency at Peak FFT Amplitude (or **Peak FFT Freq** in the text)**, iv)** Value of Peak FFT Amplitude (or **Peak FFT Amp**)**, v)** Peak Temporal Amplitude (or **Peak TEMP Amp**) and **vi)** Minimal Temporal Amplitude (or **Min TEMP Amp**).

US attenuation indicates the decrease in US intensity as it travels through the tissue, due to intrinsic effect of acoustic scattering and/or absorption. Phase Change represents the shift in phase angle of US waves, as they pass through tissue regions, characterized by different acoustic properties. Peak FFT Freq and Peak FFT Amp indicate dominant frequency component in the FFT spectrum and its amplitude, respectively; they both represent frequency characteristics of the registered US signal that has passed through tissue. Peak TEMP Amp and Min TEMP Amp refer to the highest and lowest signal intensity in time-domain waveform (McLintic and Dimech 2024).

**Figure 2.**
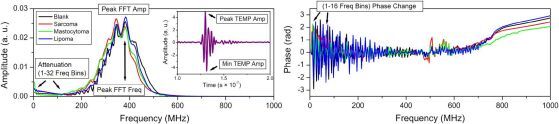
Initial analysis of US RF signals: amplitude spectrum (left panel); temporal US RF signal (left panel, inset); phase spectrum (right panel). Illustrations for calculating: Attenuation (quantified in 1–32 Freq bins, corresponding to 3.9–125 MHz frequency range) (left panel) and phase change (quantified in 1–16 Freq bins, corresponding to 3.9–62.5 MHz frequency range) (right panel); Peak FFT Freq and Peak FFT Amp (left panel); Peak TEMP Amp and Min TEMP Amp (left panel, inset).

The estimates of **Attenuation** (Figure 2, left panel) and **Phase Change** (Figure 2, right panel) were quantified using FFT amplitude and phase spectra, respectively. Both of them are evaluated relatively to the background (measurements performed on empty objective glass) and both fall within the frequency range <125 MHz, located (left) laterally to the peak of spectral characteristics curve of the US transducer (Figure 2, left panel). The range for these estimates was selected after thoroughly exploring the whole range of frequency bins. The colormaps of Attenuation and Phase Change of backscattered US signal, obtained in these frequency ranges, resulted to the sharpest visual details of the tissue structure.

The estimates of **Peak FFT Freq** and **Peak FFT Amp** were evaluated using FFT amplitude spectrum. They are both located within the range of maximal frequencies of the spectral characteristics of the US transducer (Figure 2, left panel).

The values of **Peak TEMP Amp** and **Min TEMP Amp** are obtained from the temporal domain of RF signals (Figure 2, left panel, insert). Therefore, they are completely different from previous US estimates.

In order to provide additional criterion of robustness for tissue classification, 20 (out of 512) US colormaps were collected from each US-scan along (vertical) z-axis. The criteria for image selection: i) 20 colormaps have to be consecutive; ii) temporal amplitudes have to be higher than background level; iii) colormaps are required to have the highest number of alternating (the highest and the lowest) amplitude values. According to these criteria, the sorting produced z-stacks of 20 US colormaps (Figure 13, upper panel), which all represented the internal structure of the tissue. This resulted in a large dataset: 18 cases × 10 US-scans/case × 20 images/US-scan = 3600 images for LPs. As well as, 3600 images for MCTs and 3600 images for STSs. These images were used for feature extraction and analyzed in a separate section. Each US colormap was saved separately in Tag Image File Format (TIFF).

### 2.5. Feature extraction

Computational analogs of morphological features were quantified to derive the information on the textural characteristics of the tissues (Tiwari et al. 2020), were: 1) Entropy, 2) Contrast, 3) Correlation, 4) Energy, 5) Homogeneity, 6) Average, 7) Standard deviation (st. dev.), 8) Root mean square (RMS), 9) Variance, 10) Smoothness, 11) Kurtosis, 12) Skewness.

Entropy, average, st. dev, RMS, variance, smoothness, skewness and kurtosis were computed to characterize the overall distribution of the intensity (Echegaray et al. 2018). With the aim to characterize the textural features of the image, a grayscale co-occurrence matrix was generated (Haralick et al. 1973). Subsequently, four quantitative parameters: contrast, correlation, energy and homogeneity, were calculated.

### 2.6. Classification algorithms

**Implementation of SVM.** During the initial stage of data pre-processing, the standardization of features was carried out to obtain uniformly scaled data, which is required for optimal operation of SVM classifier. SVM was arranged with a radial basis function kernel, which is well-built for detecting complex non-linear relationships by transforming input features into their respective projections, located in a higher-dimensional feature space. Other hyper-parameters were set to default values in MATLAB. SVM computes a decision boundary and evaluates the distance of each data instance from the quantified boundary in the space of higher-dimension. During the stage of post-processing, the fit Posterior function was applied. It maps the quantified distances in feature space into probabilities, which indicate the likelihood of each data point to be assigned to a particular tissue class. SVM is beneficial for the tasks of binary classification, as it effectively maximizes class-separating margin, which results in reliable generalization of the final classification output. SVM is excellent in the differentiation of complex data patterns, which can be characterized by well-defined decision boundaries (Li et al. 2018).

**Implementation of ENS.** Random Forest, a specific classifier of ENS learning, was implemented using the bootstrap aggregating (bagging) technique. This resulted into the arrangement of 100 DTs into a single classification ensemble. A single DT was trained on a subset of data, randomly sampled into training subset with sample replacement. The latter procedure corresponded to a single iteration of model learning. The eventual classification outcome of ENS model integrated the predictions of all 100 DTs by the majority voting. This specific operation of Random Forest algorithms diminishes the variability of the assignment of specific data instance to a particular class. And also, increases the robustness of the model, in the case of overfitting. Similarly to SVM, Random Forest is beneficial for operating on complex and large datasets, characterized by non-linear feature patterns. The approach of arranging multiple classifiers (DTs) into a single ensemble minimizes the possible risk of over-fitting and also improves classification efficiency making it highly reliable (Iranzad and Liu 2024). The latter features make ENS appropriate for highly heterogeneous tissue classes, such as MCT and STS.

**Figure 3.**
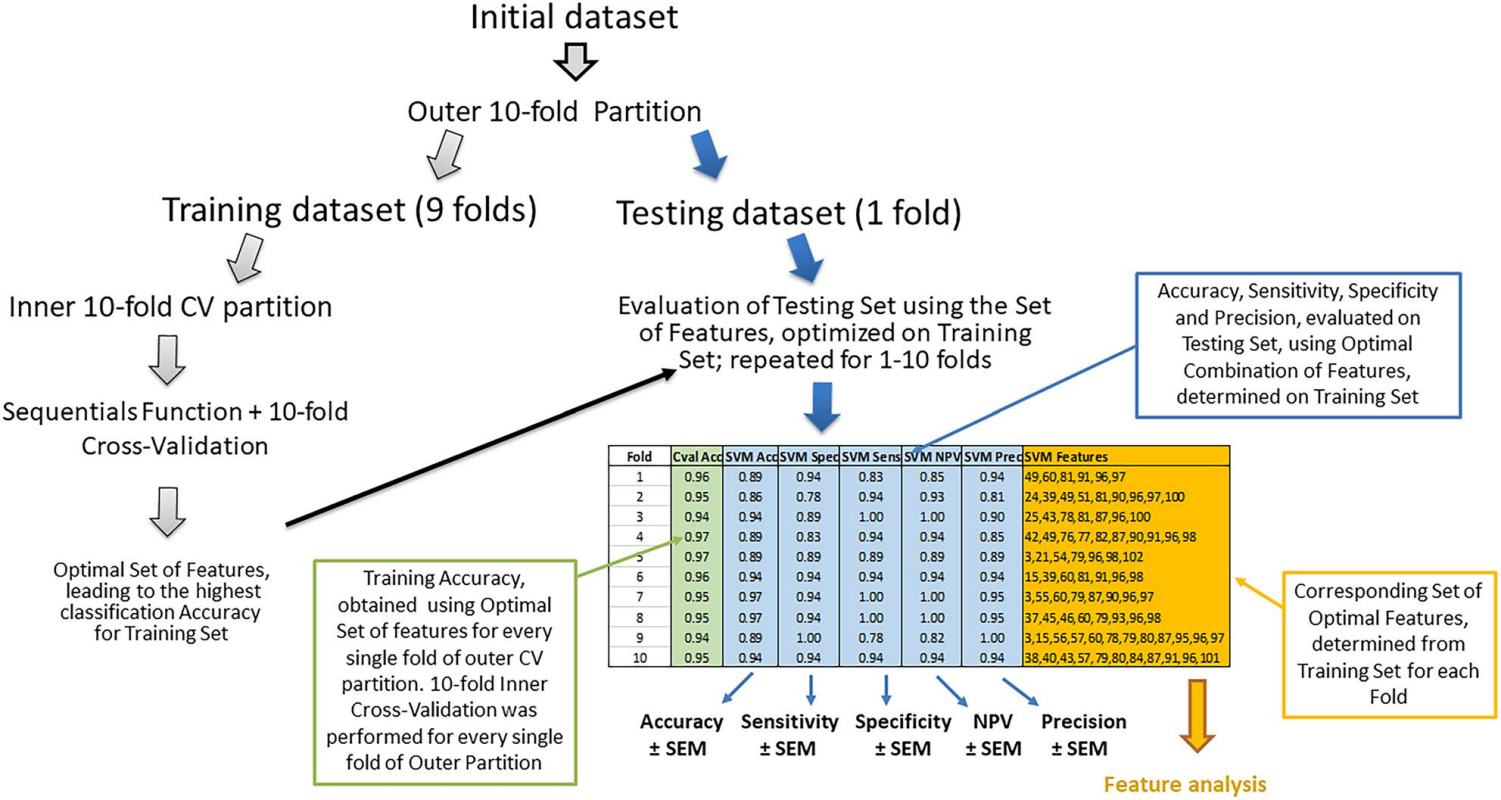
The overall dataset was split into training and testing sets using an outer 10-fold partition. Single fold was left out as unseen for the final testing of the model. Remaining 9 folds were used for the training, simultaneously coupled with the determination of optimal set of features, for every fold of the outer partition. This procedure was repeated for 1–10 iterations for each fold in the outer 10-fold partition. The obtained metrics of classification efficiency (accuracy, specificity, sensitivity, NPV and precision) were used for subsequent analysis.

**Training and Testing.** For the differentiation between MCT and STS, a set of MCT (180 image patches) and a set of STS (180 image patches) were combined into a single overall dataset, containing 360 image patches. Subsequently, it was split into training and testing sets using an outer 10-fold partition (Figure 3). Single fold, consisting of 36 image patches: 18 MCT and 18 STS, was left out as unseen for the final testing of the model. Whereas, remaining 9 folds (324 image patches: 162 MCT and 162 STS) were used for training, performed simultaneously with feature optimization. The described procedure was repeated 10 times, resulting in 10 iterations. Single iteration corresponded to each fold in outer 10-fold partition. The training/testing data split was performed randomly by setting the random number generator in MATLAB to seed #1, ensuring strict reproducibility of testing/training splits.

For the differentiation between benign and malignant tissues, the dataset of LP (180 image patches) was considered truly benign, whereas the combined dataset of MCT and STS (360 image patches) – malignant. The following procedures of training/testing were exactly the same as described above.

For the classification of MCT and STS tissues, according to US data, obtained along vertical axis, 3600 image patches of MCT were combined with 3600 image patches of STS, producing overall 7200 sample dataset for MCT and STS. For similar classification of benign and malignant tissues, the dataset of 3600 LP samples was combined with malignant (MCT and STS) dataset of 7200 samples. The following procedures for training/testing were exactly the same as described previously.

**Feature optimization.** Inner 10-fold partition was performed for 324 MCT/STS image patches, initially left for training. Sequentials function, combined with 10-fold cross-validation, was performed on training dataset to determine the optimal feature set for each fold of outer partition (Figure 3). The optimal set of features was determined according to the lowest misclassification rate (highest accuracy). The inner 10-fold split of 324 cases (to training and validation) was performed randomly by setting the random number generator to seed #1 in MATLAB, ensuring reproducibility of the results. Similar procedure was performed for the classification of benign and malignant tissues as well as for the classification, based on tissue internal structure using z-stacks of US images.

### 2.7. Evaluation of classification efficiency

#### 2.7.1. Metrics of classification efficiency

For the classification of benign and malignant tissues: benign was marked as negative (or 0), malignant – positive (or 1). Whereas for both malignant, MCT and STS: MCT – negative (or 0), STS – positive (or 1). The classification efficiency was evaluated according to (separate) primary metrics: accuracy, specificity, sensitivity, negative predictive value (NPV) and positive predictive value (PPV) or precision. Accuracy metric represents a more general estimate as it combines specificity and sensitivity into a single metric. Since combined metrics are very beneficial for multimodal analysis, for the classification of MCT and STS, we have also quantified combined secondary metric, F1 score, which integrates sensitivity and precision. This metric represents differentiation efficiency for positive class, thus, it was abbreviated as F1 score (+). Similarly, the metric, combining specificity and NPV was evaluated for negative cases, this metric was abbreviated as F1 score (−). Each metric is reported as the mean of all 10 (outer) folds ± standard error of mean (SEM). SEM represents uncertainty and implicitly addresses tissue heterogeneity, observed within the classes.

#### 2.7.2. Multimodal effect

We have strictly defined the (outer and inner) partitions of the initial dataset to be performed randomly (within 1–10 folds) (by setting, seed #1 of random number generator in MATLAB), however, identical between different groups, for example, between WL and US+WL groups. This resulted in the same combination of training and testing patches used for different multimodal groups. Therefore, we were able to directly (fold-by-fold) compare absolute values of the classification metrics between group I (WL) and group II (US+WL) as well as perform paired Wilcoxon signed rank statistical test between the imaging groups. Data analysis was performed using MATLAB (Mathworks, USA) and Origin (OriginLab Co, USA) software.

## 3. Results

### 3.1. Optical (WL and FL) imaging

**Figure 4.**
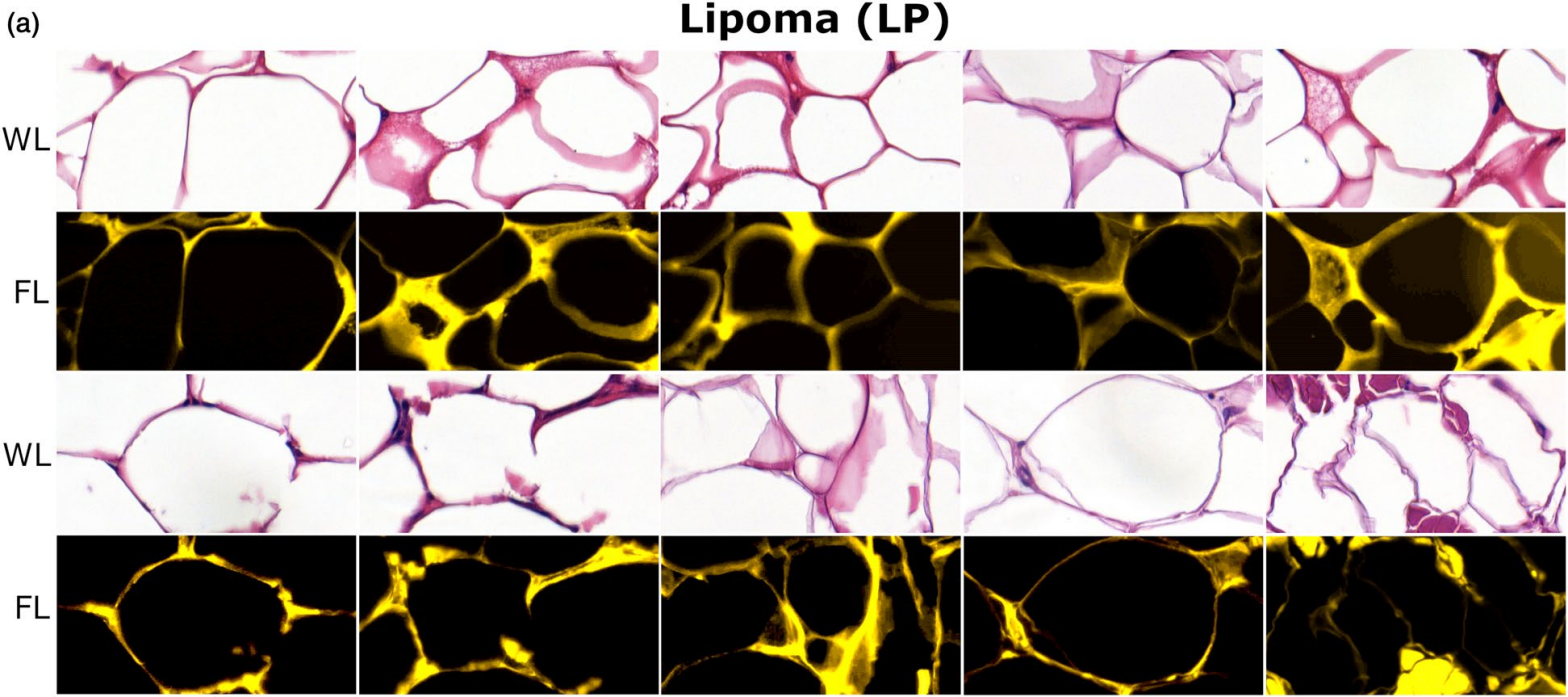
Optical (WL and FL) images of LP tissues. Mature adipocytes have clear lipid-filled vacuole and eccentric nuclei, stained in blue-purple by hematoxylin, and ECM components, stained pink by eosin (WL imaging). Collagen and elastin also exhibit strong yellow autofluorescence highlighting individual cells.

**Figure 5.**
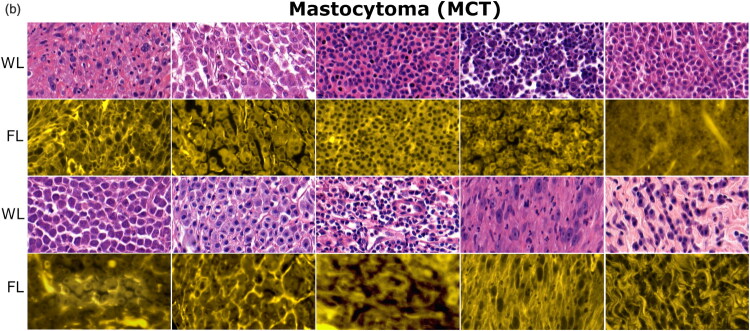
Optical (WL and FL) images of MCT tissues. Mast cells are circular-shaped with Central or slightly eccentric purple-blue nuclei. Eosin-stained cytoplasm appears faintly pink or transparent, often merged with eosin-stained ECM components (WL imaging). In particular cases (row 2, columns 2–5 and row 4, columns 1–2) FL imaging assisted in exposing individual cells. White and black stripes represent connective tissue, present in WL and FL images, respectively.

**Figure 6.**
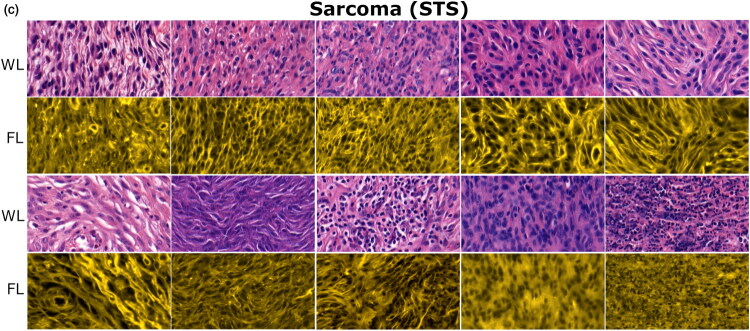
Optical (WL and FL) images of STS tissues. The majority of cells are elongated and spindle-shaped. Cell nuclei are characterized by irregular shape and size (WL imaging). FL imaging mostly highlights the stellate-shaped arrangement of ECM; over-whelming autofluorescence of ECM fibers does not allow to clearly discriminate cells.

WL and FL images, obtained for LP, MCT and STS tissues are presented in Figure 4 , 5 and 6, respectively. The number of given images is larger to adequately display the heterogeneity of MCT and STS tissues. In WL imaging, key histological features of (sub-)cutaneous LP, MCT and STS are based on their cellular composition and tissue architecture. FL imaging provides additional information on the distribution of extracellular matrix (ECM), embedding the cells.

**LP** (Figure 4). In both WL and FL images, LPs appear as a multilobular structures, reminiscent of a honeycomb. LP tissue consists of large fat-filled adipocyte cells, encapsulated by a thin net of ECM, mainly, composed collagen and elastin fibers.

**MCT** (Figure 5) and **STS** (Figure 6). WL images of malignant MCTs and STSs display a heterogeneous structure, containing varied distribution of malignant cells, ranging from sparse to dense. Cells are distributed within ECM, which varies in abundance. MCT and STS tissues differ in cell shape, which varies from round to oval for MCT and spindle to polygonal for STS, respectively. FL imaging mostly highlights collagen and elastin fibers of ECM for both malignancies. Indeed, FL imaging was very beneficial for some particular cases of MCT, because the autofluorescence of ECM fibers as well as contributing fluorescence from eosin (stains cytoplasmic proteins as well as ECM collagen and elastin) assisted in exposing individual cells (Figure 5). However, this was not that evident for STSs, because STSs, being rich in ECM fibers, exhibited strong autofluorescence, which did not allow to identify individual cells (Figure 6).

### 3.2. US imaging

**Figure 7.**
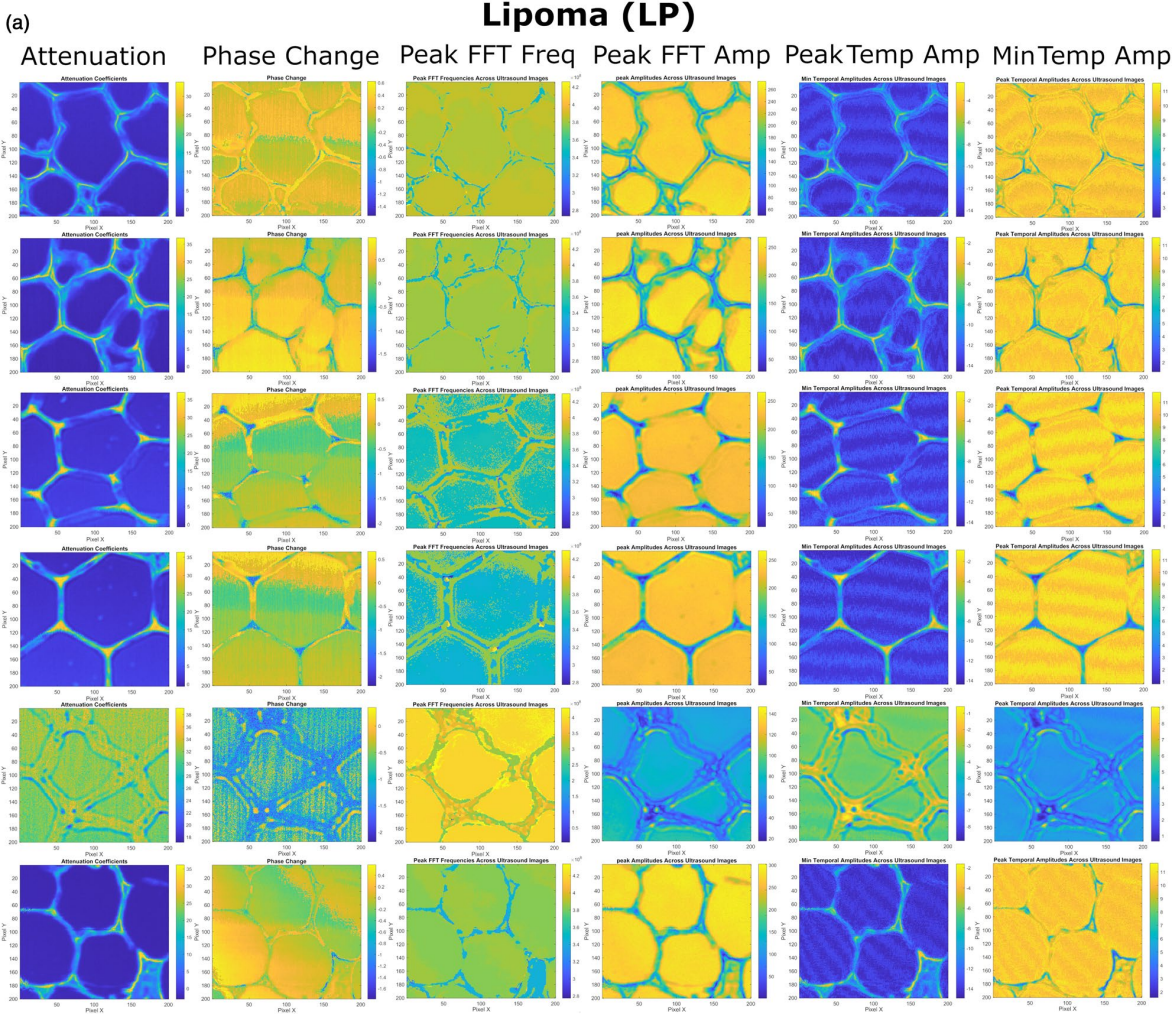
The colormaps of US spectral and temporal estimates, evaluated for LP tissues, obtained after the processing of US RF signals. Colormaps of Peak FFT Freq, presented in rows 1–2, failed to clearly expose large adipocyte cells, however, other US colormaps compensated. For the current instance, the colormaps of Peak FFT Freq, shown in rows 3–4, clearly displayed cells. In another case, overall-efficient attenuation failed to clearly identify cells for row #5, however, other estimates, especially, Peak FFT Freq – compensated.

**Figure 8.**
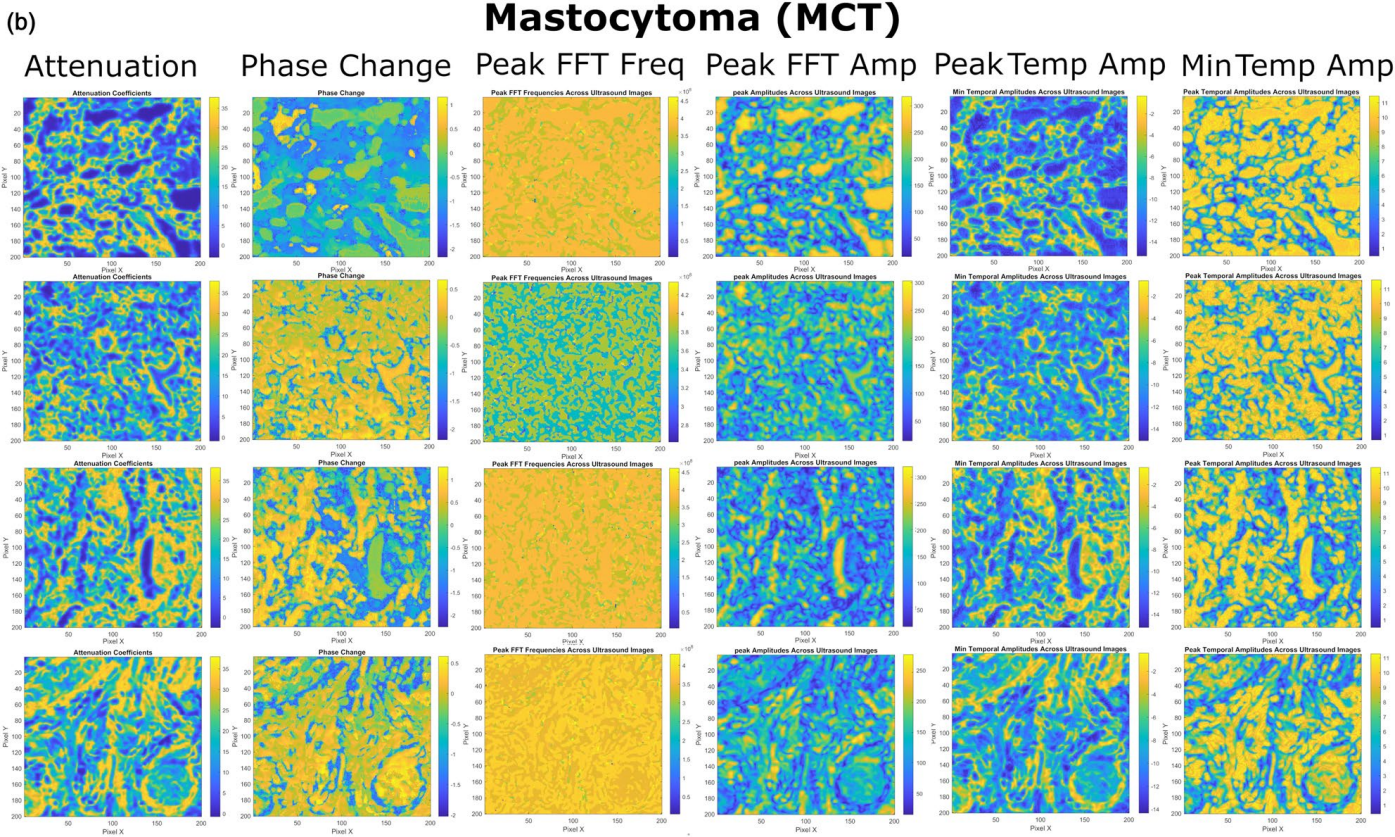
The colormaps of US spectral and temporal estimates, evaluated for MCT tissues, obtained after the processing of US RF signals. US colormaps indicate heterogeneous tissue architecture, composed of small mast cells, organized into clusters of a varied density. Different US estimates capture and highlight slightly different morphological details of cell spatial arrangement.

**Figure 9.**
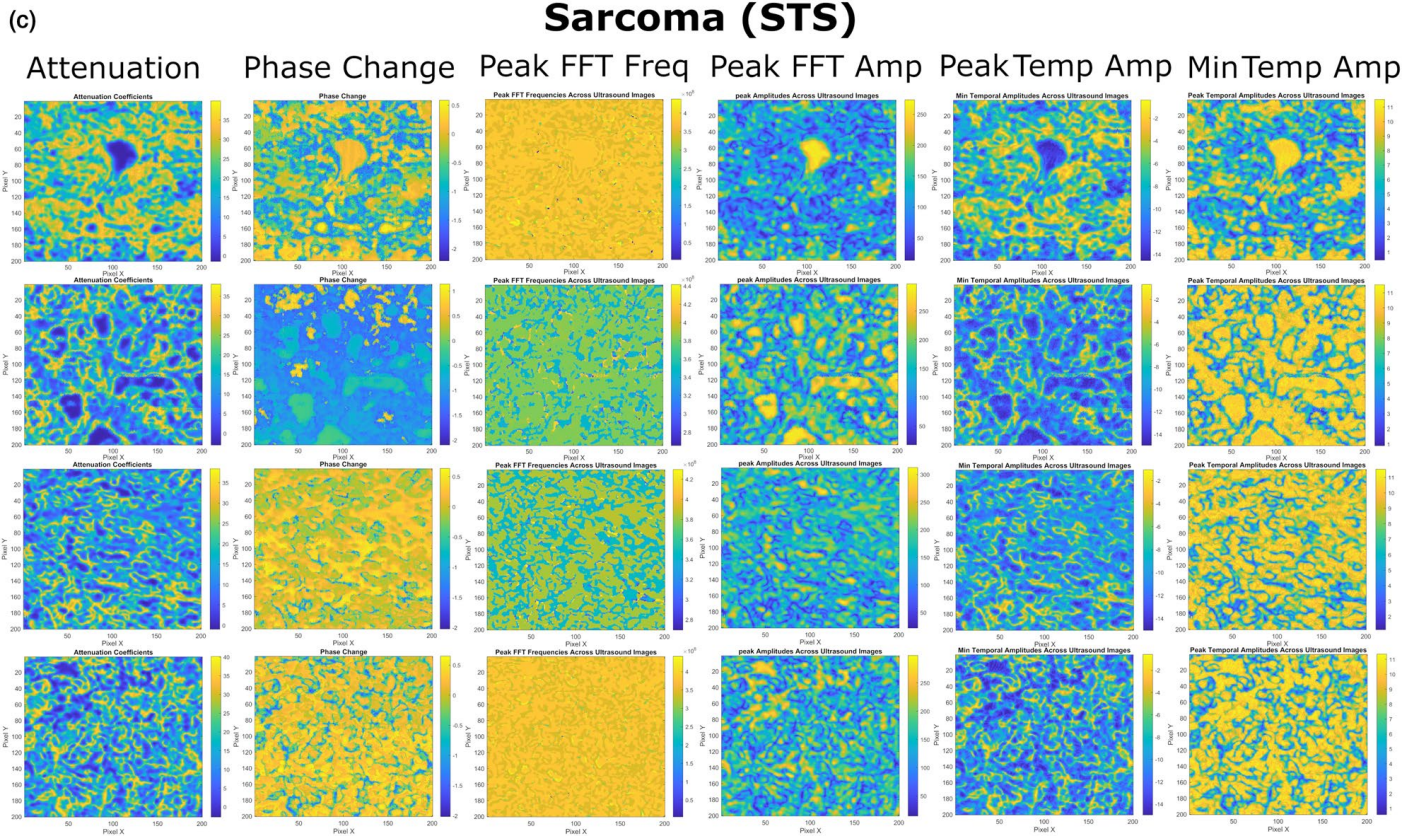
The colormaps of US spectral and temporal estimates, evaluated for STS tissues, obtained after the processing of US RF signals. STS colormaps indicate less uniform acoustic properties, representing high structural heterogeneity of the tissue. Colormaps, also, indicate higher ECM content and more regions of densely-arranged cells.

US imaging provides additional insights into the internal heterogeneity of the tumors, complementing to the optical data, which highlights tissue surface morphology. By evaluating different (spectral and temporal) domains of US RF signals, we have derived the following colormaps of US estimates: i) Attenuation, ii) Phase Change, iii) Peak FFT Freq, iv) Peak FFT Amp, v) Peak TEMP Amp and vi) Min TEMP Amp (Figures 7–9).

**LP** (Figure 7). In US colormaps, LPs exhibit typical honeycomb structure, previously observed in optical images. Different US estimates were able to highlight slightly different details of LP tissue, however, each of them was capable of capturing and displaying multilobular appearance of the tissue as well as adipocytes and surrounding ECM. If particular US estimates were unable to capture cell contours, the colormaps of other US estimates were able to compensate.

**MCT** (Figure 8) and **STS** (Figure 9). Differently from LP, US colormaps of MCT and STS tissues were identified with cells, unevenly distributed into loci, indicating areas of high and low cellular density. The morphological appearance of the tissues was variable within the colormaps, reflecting the heterogeneous nature of malignant tissues. Overall, different tissue patches of STS were identified with higher occurrence of regions of dense cellular arrangement compared to MCT.

### 3.3. Differentiation between benign and malignant tissues

**Figure 10.**
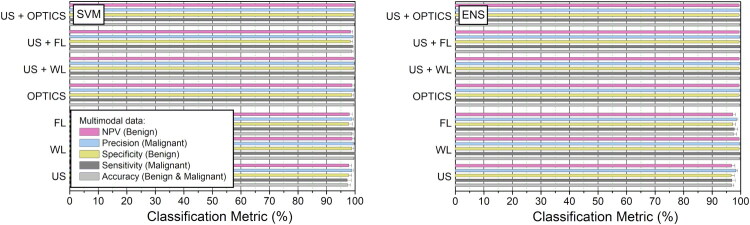
Differentiation between benign and malignant tissues for SVM (left panel) and ENS (right panel) classifiers. Classification metrics are presented for single-mode imaging: i) US (all 72 US features), ii) WL (24 features) and iii) FL (12 features); bi-modal imaging: iv) OPTICS (WL+FL) (24 WL and 12 FL features), v) US+WL (72 US and 24 WL features) and vi) US+FL (72 US and 12 FL features); as well as tri-modal imaging: vii) US+OPTICS (US+WL+FL) (72 US, 24 WL and 12 FL features). Accuracy, specificity, sensitivity, NPV and precision metrics are given after feature optimization.

Classification metrics, obtained after feature optimization, for benign (LP) and malignant (MCT and STS) tissues are presented in Figure 10.

Tissue differentiation was performed for the data of single-mode imaging: i) US, ii) WL or iii) FL data; dual-mode imaging: iv) OPTICS, v) US+WL or vi) US+FL data; and triple-mode imaging: vii) US+OPTICS. The efficiency of all classification metrics was evaluated to be in the range of 95–100% with a slightly lower percentage for single US or FL. Although, the differentiation between LP from combined set of MCT and STS tissues was trivial, and could be performed using single-mode imaging, it was essential for our classification models to be validated on well-differentiable tissues.

### 3.4. Differentiation between MCT and STS tissues

**Figure 11.**
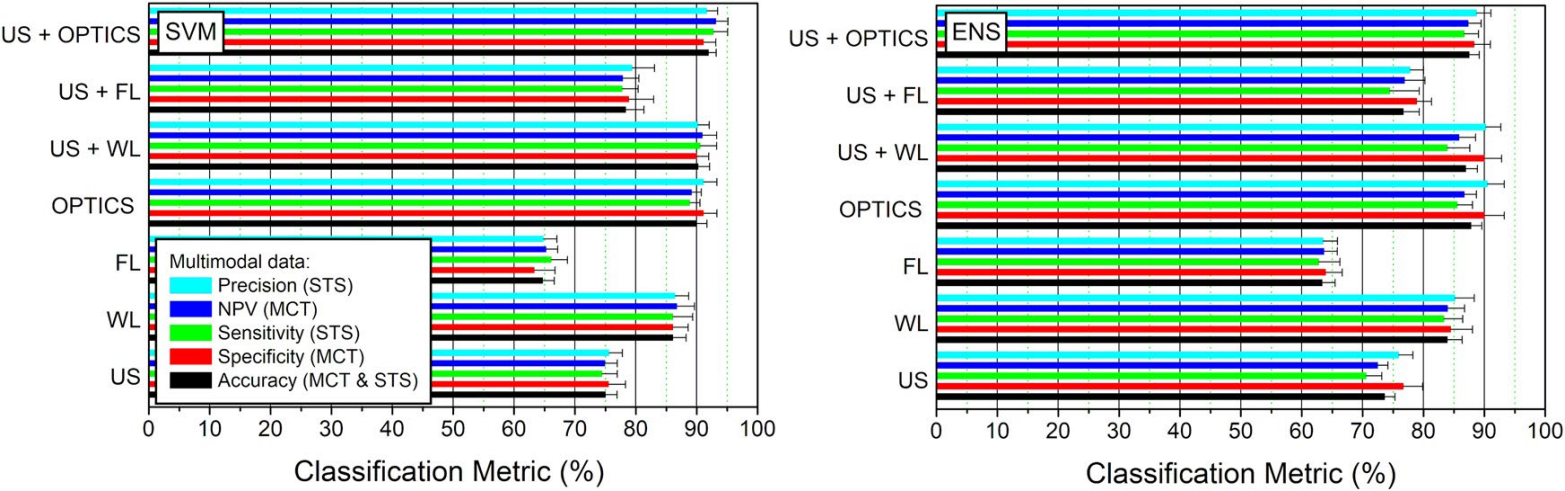
Differentiation between MCT and STS tissues for SVM (left panel) and ENS (right panel) classifiers. Classification metrics are given for single-mode imaging: i) US (all 72 US features), ii) WL (24 features) and iii) FL (12 features); bi-modal imaging: iv) OPTICS (WL+FL) (24 WL and 12 FL features), v) US+WL (72 US and 24 WL features) and vi) US+FL (72 US and 12 FL features); as well as tri-modal imaging: vii) US+OPTICS (US+WL+FL) (72 US, 24 WL and 12 FL features). Accuracy, specificity, sensitivity, NPV and precision metrics are reported after feature optimization.

Similarly to previous section, the classification of MCT and STS tissues, was performed for single, dual and triple-mode imaging. The classification metrics, evaluated after feature optimization are presented in Figure 11.

The highest diagnostic efficiency for single-mode imaging was determined for WL (∼85%), the second – for US (70–75%) and the third – for FL (∼65%) (Figure 11, left panel). However, it must be considered, that the optimization for single-mode imaging was performed using different number of initial features. Bi-modal WL+FL (OPTICS) combination was evaluated with positive increase in absolute value for all the metrics for both classifiers (up to ∼90% for SVM and ∼85–90% for ENS); US+WL – only for SVM (up to ∼90%); US+FL – for both classifiers (up to ∼80% for SVM and ∼75% for ENS) and US+OPTICS (up to ∼90–95%) – only for SVM classifier.

### 3.5. The assessment of multimodal effect

Multimodal effect was evaluated between WL and OPTICS, WL and US+WL, US and US+FL as well as OPTICS and US+OPTICS groups. Positive increment in absolute value for the classification efficiency was observed for the majority of hybrid combinations and metrics (Table 1). However, this increase in classification efficiency was statistically insignificant for the absolute majority of metrics. The evaluated multimodal increment was small (<5%) and was over-whelmed by high uncertainties, expressed as SEMs. However, a more general approach, involving statistical comparison of combined metrics, of i) accuracy, ii) F1 score (+) and iii) F1 score (−), between WL and trimodal combination of US+OPTICS, indicated significant (*p* < 0.05) multimodal effect for SVM classifier. This shows that multimodal increment from the most effective single imaging mode, WL, to triple mode of US+OPTICS, is easier to be identified as statistically significant. The data, presented in Table 1, indicates, that we have obtain increment in absolute values, when increased the number of imaging modes from one to two or from two to three, for the majority of metrics. However, this increment was identified as statistically insignificant.

The data, presented in Table 1, also indicate, that the values of classification metrics for SVM classifier have significantly increased above 90% for the group trimodal imaging. Also, uncertainties, expressed in SEMs, indicate the pronounced level of tissue heterogeneity, that was not observed for previous classification of benign and malignant tissues.

### 3.6. Analysis of selected features

**Figure 12.**
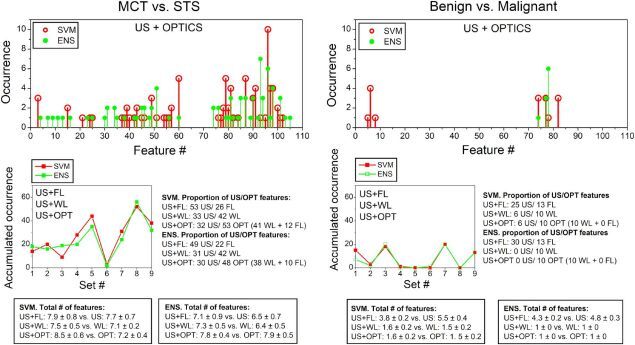
Feature occurrence (upper panel) and distribution (lower panel) for the differentiation between MCT and STS (left panel) as well as benign and malignant tissues (right panel). Upper panel indicates occurrences of single features in the generalized US+OPTICS group (optimized from overall 108 features). The lower panel indicated the summarized occurrences of features within separate sets for all multimodal groups (US+FL, US+WL and US+FL). Set numbers: 1 – Peak FFT frequency; 2 – attenuation; 3 – Peak FFT Amplitude; 4 – Peak temporal Amplitude; 5 – Minimal temporal Amplitude; 6 – Phase Change; 7 – WL1; 8 – WL2; 9 – FL. Appropriate textboxes indicate the proportion of US and optical features (combined for all 10 folds) as well as the total number of features used by the classifier within multimodal groups per single fold. The exact specification of each feature, according to its number, is given in Appendix Table A2.

The distribution of selected features in the group of US+OPTICS for the classification of MCT and STS as well as benign and malignant tissues, is presented in Figure 12. The differentiation between MCT and STS has a high variation in selected features. This was largerly dependent on specific training/testing split, since both malignant tissues were identified to be highly heterogeneous (Figure 12, left upper panel). On the contrary, for the classification of benign and malignant tissues, only a few features were required, since benign tissue was identified to be more homogeneous compared to malignant (Figure 12, right upper panel).

Figure 12 (lower panel) shows the integrated sum of feature occurrences, combined for all multimodal groups. For the differentiation between MCT and STS, both US temporal amplitudes were the most prioritized among US features. FFT estimates followed subsequently, whereas the features, derived using US Phase Change colormaps, were completely ignored (Figure 12, left lower panel). Although, the optimization function strongly preferred WL set for both classifiers, FL features were also popular even in rich US+OPTICS dataset. This was surprising, since US+OPTICS dataset already possessed many features of WL and US. The latter were evaluated to be superior to FL, since single FL imaging was not determined to be that efficient (Figure 11). In total, a relevant number of US features was selected for SVM and ENS classifiers in multimodal groups. This indicates that US features were, indeed, competitive with optical counterparts, especially in combination with FL. Although, the features of FL, were determined to be inferior to US, if tested alone (Figure 11), they were successfully added to superior US features for ENS classifier or successfully replaced optical counterparts for SVM classifier.

For US+WL and US+OPTICS, the importance of US features decreased, since WL features were determined to be superior to US features for tissue differentiation. However, still, the proportion of US/optical features was 33/42 and 32/53 for SVM as well as 31/42 and 30/48 for ENS (Figure 12, left lower panel) for the differentiation between MCT and STS in US+WL or US+OPTICS groups, respectively. Also, the total number of optimal features did not markedly increase in US+WL or US+OPTICS groups, compared to WL or OPTICS (Figure 12, left lower panel). This indicates that US features were selected by the optimization function to be added to or to replace superior WL features.

Not surprisingly, US and FL features were over-whelmed by WL counterparts for the differentiation between benign and malignant tissues (Figure 12, right lower panel). For SVM, only 1–2 total features were required to differentiate LPs from malignant tissues. Whereas, for ENS – only single feature from WL set – the homogeneity was preferred 3 times and the average − 6 times.

General tendencies in feature selection:

1. for the differentiation between benign (LP) and malignant (combined MCT and STS) tissues, classifiers preferred features, obtained from WL imaging; additionally it required neither US nor FL features. However, if WL imaging mode was absent, both US and FL features were required.
2. On the contrary, for the classification of MCT and STS tissues, classifiers required the combination of US, FL and WL features to a varied proportion, which can be attributed to the heterogeneity of both MCT and STS tissues.

### 3.7. Tissue classification according to internal structure

**Figure 13.**
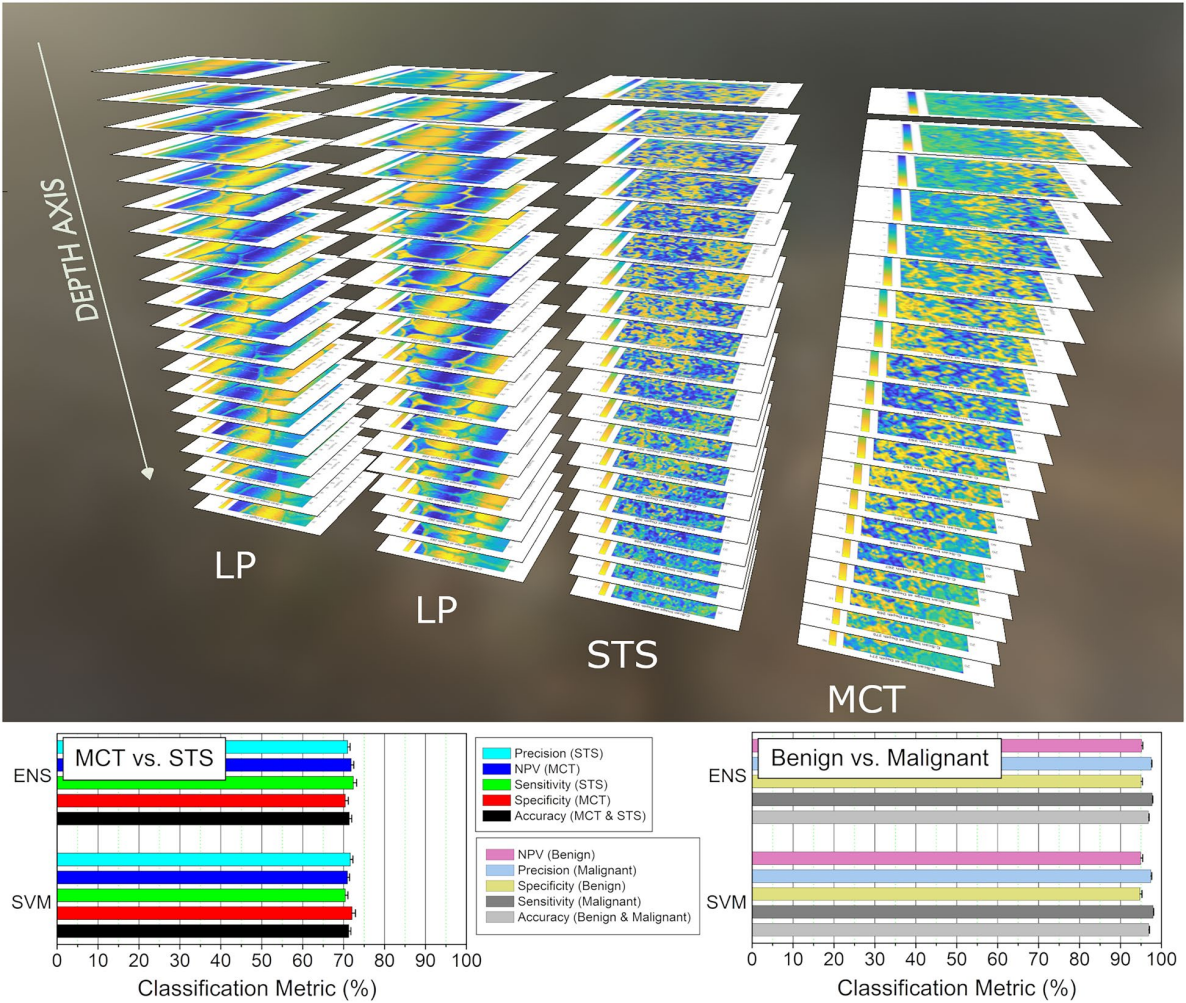
Z-Stacks of tissue colormaps, obtained along the vertical (scanning) axis of US 3D arrays of RF signals. 20 consecutive tissue images, representing US temporal amplitude along the vertical axis, were obtained for each selected locus within the histological slide (upper panel). Classification metrics, evaluated for MCT and STS (lower left panel) as well as benign and malignant tissues (lower right panel), are reported after feature optimization (12 US features).

We have already analyzed the morphological features, derived from spectral and temporal data of US imaging. However, we have only employed the information, provided by peak or min temporal US amplitudes. Since, US C-scans provide US colormaps at each step in the vertical (scanning) axis, additional classification was performed using z-stacks of US colormaps. For this purpose, 20 consecutive tissue images, displaying the internal structure of the tumor, were selected from each US C-scan, (Figure 13, upper panel).

The classification resulted in ∼70% efficiency for MCT and STS as well as >95% for benign and malignant tissues (Figure 13, lower panel). Current differentiation was performed completely on the US imaging data, representing internal tissue structure. The obtained level of classification efficiency can be reported as a secondary criterion, representing the robustness of a classifier and used to indicate the level of additional reliability of clinical diagnosis for heterogeneous tumors.

## 4. Discussion

In current study, tissue slides of canine/feline LP, MCT and STS tumors were examined using US, WL and FL imaging modalities. Relevant US colormaps and optical images were produced for each imaging modality. Next, AI algorithms were applied for the classification of cancer tissues with the aim to establish a differential diagnosis. Quantified equivalents of morphological features were optimized *via* sequential feature selection, coupled with 10-fold cross-validation. Eventually, classification algorithms were validated on unseen (left-out) dataset. While other studies in the field of veterinary oncology have already investigated multimodal diagnostics (Li et al. 2020; Tiwari et al. 2020; Tamošiūnas et al. 2022; Dank et al. 2023), our study introduced two main advancements:

- a cohesive integration of US, WL and FL imaging into innovative bi-modal and tri-modal hybrid approaches, leading to enhanced classification efficiency;
- a detailed statistical analysis of primary and secondary classification metrics, leading to significant multimodal effect identified for tri-modal US+OPTICS combination;
- implementation of sequential feature addition, aiding in correct interpretation of multimodal effect; as the features, obtained via subsequent imaging modes are not simply pooled together, but, rather, automatically selected by the optimization algorithm;
- extensive feature analysis, expanding the conventional understanding of multimodality: i) features, obtained via different imaging modes can be selected by the automated algorithm to be added together; or ii) features, obtained via the second imaging mode may be selected to substitute the features, proved by the primary mode.

For the classification of benign and malignant tissues we have achieved almost 100% classification efficiency for both SVM and ENS classifiers for all the imaging modes or their combinations. Also, we were able to discriminate LPs from malignant tumors (at ∼100% efficiency) using only single WL feature for ENS classifier and 1–2 US/optical features using SVM classifier. These features were the average and the homogeneity for the absolute majority of cases.

Subsequently, we put more focus for the differentiation between two heterogeneous tissues, MCT and STS. This resulted in the following classification efficiency: up to 75% for US; 75–80% for US+FL; 85–90% for US+WL and 87–93% for US+OPTICS (Figure 11). The differentiation between MCT and STS tumors, characterized by heterogeneous tissue structure but different pathogenesis, is considered to be one of the major challenges, encountered in veterinary oncology. Currently achieved high (>90%) classification efficiency for SVM can be considered as a significant advancement not only over pioneering single-mode imaging studies, but also other ML-based diagnostic studies, performed in the field (Kircher et al. 2012; Mesa et al. 2017; da Cruz et al. 2022; de Nardi et al. 2022; Tamošiūnas et al. 2022; Dank et al. 2023). Our conceptual approach presents several methodological and computational improvements. First, while pioneering studies relied ML models, implemented on single-mode datasets, such as US imaging (da Cruz et al. 2022) or OCT (Mesa et al. 2017), our strategy employes the combination of US, WL and FL modalities. Second, the majority of studies, utilized large datasets of fixed feature numbers, or employed deep learning algorithms, that were trained on entire tissue patches, thus, losing valuable interpretation (Kircher et al. 2012; de Nardi et al. 2022; Tamošiūnas et al. 2022; Dank et al. 2023). On the contrary, we have employed sequential feature addition, which successively creates a set of optimal morphometric parameters, hereby, increasing classification robustness and diminishing redundancy.

Based on our previous works, we have employed SVM classifier, as it was the most efficient in providing the differential diagnosis on melanomas (Tiwari et al. 2020). In addition, we have applied Random Forest classifier from the group of ENS learning. Both classifiers are naturally nonlinear, and, as a result, they can detect and identify non-linear relationships between sophisticated feature patterns. Our results indicate that both SVM and ENS classifiers are similar in the efficiency in differentiating between benign and malignant as well as MCT and STS tumors. Although, in general, ENS may be considered superior in terms of robustness and generalizability (Li et al. 2018; Iranzad and Liu 2024), we found that SVM was more efficient in detecting multimodal effects (Figure 11).

The cohort, used in our study is, relatively, moderate, consisting of 51 animals. However, all of them were clinically-indicated cases. Every tumor was meticulously analyzed to create a dataset of high-resolution image patches (180 per tumor class), which increased the robustness of ML algorithms. The number of datapoints was markedly increased by applying the data arrangement strategy of patch-level-splitting. However, this was unable to account for tumor biological diversity, associated with age, breed and grade variations. However, study cohort included both canine and feline individuals across an extensive range of age (2–13 years), as well as anatomically diverse tumor origination sites, which partially account for variability, observed in real world.

Due to moderate number of animals, we were restricted to patch-level sampling, where multiple patches from the same tumor appear in both training and testing sets. This rationale was intentional, as it allowed to capture full spatial intra-tumoral heterogeneity by creating rich and diverse dataset. It was consistent with our aim, to determine, whether biophysical features can efficiently distinguish malignancies in multimodal approaches. Since, we did not intend to derive a diagnosis on a single patient level and, because each patch was a real and meaningful subunit of histological information, we considered patch-level data arrangement as appropriate.

Indeed, by addressing intra-tumoral heterogeneity, patch-level splitting allowed to mimic local diagnostic uncertainties. The latter are unavoidable in real-world histopathological occasions, where the identification of ambiguous heterogeneous loci is one of the core issues. Also, this strategy provided sufficient data variability, required for feature optimization. Furthermore, it diminished the risk of possible under-fitting, which becomes a concern when patient-level splitting is performed on a small sample size. Random patch-level splitting also reduced the risk of unique subset, left for model validation. This could result in the failure of model fitting, again, introducing another ambiguity. We acknowledge, that compared to real-world scenario, reported classification metrics may vary. However, in the current study, we were mostly focused on detecting positive multimodal increment between combinations of imaging modes. And also, provide a proof-of-concept, which is essential prior to moving toward patient-level splitting.

Possible over-estimating of classification efficiency, mostly influenced by small sample size, was diminished by employing advanced data processing strategies. SVM classifier is, in general, designed to perform on smaller datasets, whereas ENS was composed of 100 DTs to increase the robustness. Classification accuracy was tested on 10 independent random data splits, ensuring that no unique dataset was left out and subjected for eventual testing. The study, also, reports a meticulous error analysis, indicating the confidence levels for metric reliability. A valuable novelty is constant training/testing data partition, associated with concrete iteration number. This allows to detect even the slightest multimodal increment using paired statistical test. Additionally, possible over-fitting was mitigated by the integration of sequential feature addition, applied simultaneously with inner cross validation. This kept the number of utilized features significantly lower compared to patch number. Lastly, there is a plethora of research studies, utilizing ML tools, which well-perform on larger number of cases. However, in the near future, the models that effectively perform on small datasets, may be favored and prioritized. Therefore, the concept and implementation of our algorithm, presented in Figure 3, may be perceived as a promising innovation in the field.

Our current approach is not simply training the model on 12 US features, later – on 12 optical features and then on combined 24 US and optical features, compared to previous simplified approaches (Tiwari et al. 2020; Tamošiūnas et al. 2022). This may introduce a discrepancy in the interpretation/confirmation of multimodal effect, because the classifier is simply trained on a larger number of features. In our study, we have employed sequential feature selection, this optimization ensured that only relevant features were selected. We have used similar number of total features for single as well as multimodal combinations for the classification of MCT and STS. Thus, the multimodal effect could be properly addressed and interpreted, avoiding possible ambiguities. Additionally, high differentiation efficiency between benign and malignant tissues, using only 1–2 optimal features, demonstrated that high accuracy can be attained with minimal set of data.

Furthermore, we have performed 10-fold outer data partition to training/testing subsets to properly address the classification efficiency in dependence on data variability. We have not relied only on simple reporting of classification metrics, obtained from a single or multiple independent confusion matrices, without considering uncertainties. Therefore, our study provides detailed insights into primary (accuracy, specificity, sensitivity, NPV and precision) and secondary [F1 score (+) and F1 score (−)] classification metrics with the evaluation of errors. This leads to a deeper knowledge in the heterogeneous nature of MCT and STS tumors and enhances classification reliability, simultaneously, indicating diagnostic limitations. Unfortunately, we have not obtained statistically significant multimodal effect, when increasing the number of imaging modes from one to two or two to three. However, we achieved relevant multimodal effect (*p* < 0.05) for combined metrics of accuracy, F1 score (+) and F1 score (−) when increasing the number of imaging modes from one to three. What is more, our study demonstrates that inferior features of US can be added or substitute superior WL features – a phenomenon, that can also be considered as a manifestation of positive multimodal effect, even though, classification efficiency remains similar.

Multimodal imaging, such as US+WL or US+OPT, has led to the increment in absolute values of the majority of classification metrics. However, only a few of them were evaluated to be statistically significant. This could be attributed to heterogeneous nature of MCT and STS tissues, introducing higher uncertainties to primary metrics. This goes line with insignificant multimodal increment, determined for single vs. bi-modal or bi-modal vs. tri-modal imaging approaches. However, significant metric increase was evaluated for the accuracy metric during the comparison of single and tri-modal imaging approaches for SVM classifier. This may be associated to the combined nature of accuracy metric, which incorporates two primary metrics, specificity and sensitivity, resulting in less-pronounced variability. Also, specific metrics are related to particular tumor class, for example, sensitivity and specificity represent correct identification of STSs and MCTs, respectively. For instance, as described in section ‘2.1’, in contrast to STS, FL imaging highlighted more details in some MCT patches. However, this may result in both specificity and sensitivity increase, since MCTs are more efficiently discriminated from STSs, but also STSs may be better differentiated from MCTs. The data presented in Table 1, indeed, indicates the tendency of specificity or sensitivity increase for US→US+FL or WL→OPT groups, however, this increment is observed only in the absolute value of the metrics.

Up to date, the most significant research progress has been made using OCT for image-guided detection in veterinary oncology. Due to providing insights in specific microstructural appearance, OCT enabled to detect feline sarcoma (Mesa et al. 2017; Coleman et al. 2021), canine mammary (Fabelo et al. 2021) and canine STS (Selmic and Ruple 2020; Lages and Selmic 2021) tumors and their boundaries. However, OCT has certain limitations, such as challenging OCT image interpretation and a few millimeter penetration depths, which restrain its applications. Therefore, OCT has been integrated with Raman spectral band imaging (Tamošiūnas et al. 2022) as well as magnetic resonance imaging or photoacoustic microscopy (Kircher et al. 2012). However, these are high-cost cutting-edge devices, generally unavailable in conventional veterinary facilities. Therefore, we propose to employ accessible optical imaging (WL or FL) in tandem with US as an alternative. US provides information on internal tissue structure, WL – high resolution surface morphology, and FL – highlights the components of ECM and/or cell-to-cell contrast. Currently, we have obtained an increment in diagnostic efficiency for both classifiers, when combining US and optical techniques for the differentiation between MCT and STS tissues. This implies that different data may be employed for the discrimination between heterogeneous tissues. In addition, we have introduced a secondary criterion to evaluate the classification efficiency according to the information on tissue internal structure, obtained using US RF signals. Therefore, the accuracy of SVM for the differentiation between MCT and STS tissues (for US+WL group) can be expressed as: 90.3 ± 1.9% (US+WL)×71.2 ± 0.6% (US_Depth Profile_). This could introduce additional reliability for the diagnostic method and be beneficial for the classification of heterogeneous tissue classes. Although, US+WL already had the information on tissue internal properties, however, the information from US time-domain had been represented only by peak or minimal amplitude. Therefore, the diagnostic information of US imaging is not used to its full capacity. In regular veterinary clinics, expensive US C-scan may be replaced by economical and more available B-scan systems. This imaging strategy has also been successfully employed for the diagnostics of much smaller human skin tumors (Tiwari et al. 2020).

We acknowledge, that: i) histopathological identification has been performed for all the tumors, used in the study, and ii) the image patches were selected within the loci of cancerous tissue, delineated by experienced veterinary histopathologist. Thus, the obtained classification metrics should not be perceived as indicators of clinical diagnostic efficiency. Rather, they should be interpreted as the measures of internal ML-based classification performance, determined on pre-labeled data. Current study serves as a proof-of-concept, representing the synergistic potential of multimodal imaging and AI techniques, to provide valuable assistance to veterinary oncologists. We also recognize that cytology and histopathology are gold standards of veterinary diagnostics, that cannot be replaced even by cutting-edge imaging machinery.

However, the diagnostic output, provided by ML algorithms strongly correlated with human-derived diagnoses in different biomedical approaches (Ehteshami Bejnordi et al. 2017, 2018; Nir et al. 2019; Hägele et al. 2020; Kanan et al. 2020; Massion et al. 2020; da Silva et al. 2021; Faryna et al. 2024). Therefore, the modalities of US and optical imaging, which represent physical quantities, obtained at the tissue level, may be, indeed, valuable for some complex cases or cancer types. Although, the tissue evaluations, attained on the biophysical level, are far from being competitive replacements for histological diagnostics, they may provide a valuable diagnostic backup for veterinary specialists. For instance, the employment of US imaging, at the level of RF signal analysis, provides an information on the internal tissue structure. Alternatively, FL imaging highlights the arrangement of the components of ECM, that cytology cannot capture. Thus, the proposed method is developed with the aim to complement conventional diagnostic techniques, that could become very useful when histology or biopsy results are ambiguous. Therefore, our study aims to provide an accurate and automated method for the differentiation of (sub-)cutaneous tumors in real-time. This could become a reliable support for veterinary histologists for the double-check of diagnostic accuracy when working with tissue slides and performing histological evaluation. In particular, it is very beneficial for the challenging differentiation between MCTs and STSs, which can be double-examined, if ML prediction contradicts with human-derived diagnosis.

Our study was conducted on histological slides, which are a standard research model. Current *ex vivo* design is excellent for proof-of-concept studies, as it allows for high-resolution imaging and reproducible feature extraction. However, it is very different from freshly-resected *ex vivo* samples or complex *in vivo* conditions. Although, the complex milieu of living tissue strongly impedes high quality imaging, a framework of our diagnostic concept may be adopted into veterinary practice in the long term. Indeed, clinical feasibility of our method would require only minor adjustments from routine procedures. The workflow would involve a veterinary specialist, collecting the set of WL and FL images on tissue slides, as well as scanning the resected tumor with an echoscope. Tissue images may be directly uploaded to computer, installed with ML-based application. After feature quantification, the currently extracted feature set would be compared with large database of references, that had been used for classifier training. The latter technology would provide a supplementary decision-support to conventional techniques, offering a second opinion or double-check option before treatment.

In the end, we recognize several limitations of our study, that require to be addressed. Due to low study cohort, we were restricted to a patch-level strategy of data arrangement. Therefore, the next step would be the implementation of patient-level analysis after collecting more animal samples. Secondly, current research was conducted on *ex vivo* histological samples, subsequent employment of our concept into *in vivo* platform will demand the adjustment of imaging protocols and additional validation. Third, the research was performed using high-resolution SAM and FL imaging, which may not be available in standard veterinary clinics. However, simplified versions of our concept may be implemented using US B-scans and standard optical setups. Finally, further studies will focus on: expanding animal cohort, incorporating tumor grade and, in long term, translating form *ex vivo* to *in vivo* settings.

In conclusion, we present a state-of-the-art diagnostic framework, integrating US, WL and FL imaging modes into hybrid trimodal combination. Diagnostic method is specifically designed for the analysis of histological tissue slides of canine/feline (sub-)cutaneous tumors and allows to achieve high classification efficiency (>90%). The reproducible performance of ML-based classification algorithms suggests that our methodological concept is capable to identify essential differences, which are robust at tissue patch-level split. Current model could be beneficial for veterinary laboratories, which already work on histological slides. The method aims to provide an automated real-time support, so that ambiguous cases would receive a repeated inspection, performed by veterinary histologist.

## AI transparency

An AI-generated image of a hypothetical sarcoma-bearing cat is presented in Figure 1 for illustrative purposes. The image was created using OpenAI’s DALL-E. We also acknowledge the assistance of OpenAI’s ChatGPT in refining the English language of this manuscript.

## Supplementary Material

Supplements.docx

## Data Availability

The datasets generated and/or analyzed during the current study are available from the corresponding author upon reasonable request.
