# Supplementary material for: Machine learning based diagnostics of veterinary cancer on ultrasound and optical imaging data
Source: Vet Q. 2025 May 30;45(1):1–19. doi: 10.1080/01652176.2025.2510486 (PMC12493615; doi:10.1080/01652176.2025.2510486)
Supplement: Supplements.docx [file TVEQ_A_2510486_SM8448.docx]

**APPENDICES**

**Appendix_Table_Study_Cohort.** Detailed description of the cases of (sub-)cutaneous tumors

| **#** | **NUMBER OF SAMPLE** | **SPECIES** | **BREED** | **AGE, y/o** | **TUMOR** | **GRADE OF MALIGNANCY** | **LOCALIZATION** |
| --- | --- | --- | --- | --- | --- | --- | --- |
| 1 | 20B/0273 | Dog | Chi-hua-hua | 3 | Lipoma | - | Subcutis, shoulders |
| 2 | 20B/0383 | Cat | Sphynx | 7 | Lipoma | - | Subcutis, hind leg |
| 3 | 20B/0402 | Cat | Mix | 12 | Fibrolipoma | - | Subcutis, hind leg |
| 4 | 20B/0424 | Dog | Rottweiler | 5 | Fibrolipoma | - | Subcutis, front leg |
| 5 | 20B/0509 | Dog | Labrador | 5 | Lipoma | - | Subcutis, base of tail |
| 6 | 20B/0285 | Dog | American Staffordshire terrier | 12 | Lipoma | - | Subcutis, left axilla |
| 7 | 20B0707 | Dog | Flandrian bouvier | 11 | Lipoma | - | Subcutis, right flank |
| 8 | 20B0817 | Dog | Rottweiler | 6 | Lipoma | - | Subcutis, mammary gland |
| 9 | 20B0831 | Dog | American Staffordshire terrier | 6 | Lipoma | - | Subcutis, right hind leg |
| 10 | 20B0845 | Dog | Golder retriever | 11 | Lipoma | - | Subcutis |
| 11 | 20B0847 | Dog | Labrador retriever | 7 | Lipoma | - | Subcutis, left flank |
| 12 | 20B0879 | Dog | Jack-russel terrier | 6 | Lipoma | - | Subcutis, left axillary region |
| 13 | 20B0896 | Dog | long hairedcollie | 7 | Infiltrative lipoma | - | Subcutis, muscles, abdominal wall |
| 14 | 20B0029 | Dog | French bulldog | 11 | MCT | High grade of malignancy | Skin, preputium |
| 15 | 20B0055 | Dog | Pug | 8 | MCT | Index of mitosis 2 | Subcutis, chest |
| 16 | 20B0066 | Dog | Dachshund | 5 | MCT | High grade of malignancy | Neck |
| 17 | 20B0072 | Dog | Jack-Russel terrier | 6 | MCT | Low grade of malignancy | Skin, scrotum |
| 18 | 20B0135 | Cat | Mix | 10 | MCT | n/a | Skin, shoulders |
| 19 | 20B0150 | Dog | Golden retriever | 3 | MCT | Low grade of malignancy | Skin, shoulders |
| 20 | 20B0270 | Dog | Staffordshire terrier | 9 | MCT | Low grade of malignancy | Skin, back (x2) |
| 21 | 20B0310 | Dog | Jack-Russel terrier | 11 | MCT | Low grade of malignancy | Skin, scapular region |
| 22 | 20B0318 | Cat | Sphynx | n/a | MCT | n/a | Skin, neck |
| 23 | 20B0347 | Dog | Cane corso | 5 | MCT | Index of mitosis <1 | Subcutis, frontal region |
| 24 | 20B0446 | Dog | Retriever | 7 | MCT | Low grade of malignancy; 2 grade | Skin, metatarsal region |
| 25 | 20B0516 | Dog | French bulldog | 9 | MCT | Low grade of malignancy | Skin, ear |
| 26 | 20B0544 | Dog | American bulldog | 9 | MCT | Low grade of malignancy | Skin, hind leg |
| 27 | 20B0657 | Cat | Mix | 6 | MCT | Low grade of malignancy | Skin, mammary glands |
| 28 | 20B0935 | Dog | American Staffordshire terrier | 4 | MCT | Low grade of malignancy | Skin, ventral part of chest |
| 29 | 20B0016 | Cat | Mix | 12 | STS | III | Skin, base of tail |
| 30 | 20B0041 | Cat | Mix | 13 | Anaplastic sarcoma | III | Subcutis |
| 31 | 20B0422 | Dog | Cane corso | 8 | Anaplastic sarcoma | III | Skin, scapular region |
| 32 | 20B0435 | Cat | Mix | 11 | Anaplastic sarcoma | n/a | Subcutis, scapular region |
| 33 | 20B0489 | Dog | Jack-Russel terrier | 10 | STS | II | Skin, elbow |
| 34 | 20B0493 | Cat | Persian | 11 | Fibrosarcoma | II | Shoulder |
| 35 | 20B0507 | Dog | Mix | 2 | Hemangio- sarcoma | n/a | Skin, subcutis, knee |
| 36 | 20B0543 | Dog | Mix | 10 | STS | II | Skin, elbow |
| 37 | 20B0493 | Cat | Persian | 11 | Fibrosarcoma | II | Shoulder |
| 38 | 20B0614 | Dog | Labrador | 3 | STS | III | Subcutis, elbow |
| 39 | 20B0621 | Dog | Mix | 12 | STS | II | Hind leg |
| 40 | 20B0623 | Cat | Mix | 11 | STS | II | Subcutis, chest |
| 41 | 20B0614 | Dog | Labrador | 3 | STS | III | Subcutis, elbow |
| 42 | 20B0649 | Dog | Mix | 12 | STS | III | Subcutis, perianal region |
| 43 | 20B0647 | Dog | Mix | 6 | STS | II | Skin, subcutis, right axillary region |
| 44 | 20B0552 | Dog | Bernese shepherd | 9 | Mixed liposarcoma | II | Ear |
| 45 | 20B0679 | Cat | Mix | 13 | Fibrosarcoma | III | Subcutis, left hind leg |
| 46 | 20B0662 | Dog | Russian – European laika | 10 | STS | II | Skin, subcutis, left anconeus region |
| 47 | 20B0776 | Cat | Mix | 11 | Fibrosarcoma | II | Subcutis, between shoulders |
| 48 | 20B0782 | Dog | Mix | 8 | Hemangio- pericytoma | I | Skin, subcutis, left elbow region |
| 49 | 20B0835 | Cat | British short hair | 9 | Fibrosarcoma | II | Subcutis, between shoulders |
| 50 | 20B0860 | Cat | Mix | 11 | STS | II | Skin, subcutis, chest region |
| 51 | 20B0868 | Cat | Mix | 5 | Fibrosarcoma | II | Subcutis, caudal part of back |

**Appendix_Table_Feature_Description.** The numbers for each morphological feature within each US, WL and FL set.

| **FEATURE**  **GROUP** | E  N  T  R  O  P  Y | C  O  N  T  R  A  S  T | C  O  R  R  E  L  A  T  I  O  N | E  N  E  R  G  Y | H  O  M  O  G  E  N  E  I  T  Y | A  V  E  R  A  G  E | S  T.    D  E  V | R  M  S | V  A  R  I  A  N  C  E | S  M  O  O  T  H  N  E  S  S | K  U  R  T  O  S  I  S | S  K  E  W  N  E  S  S |
| --- | --- | --- | --- | --- | --- | --- | --- | --- | --- | --- | --- | --- |
| **ULTRASOUND DATA** | | | | | | | | | | | | |
| US PEAK FFT FREQUENCY | **1** | **2** | **3** | **4** | **5** | **6** | **7** | **8** | **9** | **10** | **11** | **12** |
| US ATTENUATION | **13** | **14** | **15** | **16** | **17** | **18** | **19** | **20** | **21** | **22** | **23** | **24** |
| US PEAK FFT AMPLITUDE | **25** | **26** | **27** | **28** | **29** | **30** | **31** | **32** | **33** | **34** | **35** | **36** |
| US PEAK TEMPORAL AMPLITUDE | **37** | **38** | **39** | **40** | **41** | **42** | **43** | **44** | **45** | **46** | **47** | **48** |
| US MINIMAL TEMPORAL AMPLITUDE | **49** | **50** | **51** | **52** | **53** | **54** | **55** | **56** | **57** | **58** | **59** | **60** |
| US PHASE CHANGE | **61** | **62** | **63** | **64** | **65** | **66** | **67** | **68** | **69** | **70** | **71** | **72** |
| **OPTICAL DATA** | | | | | | | | | | | | |
| WHITE LIGHT SET #1 (WL 1) | **73** | **74** | **75** | **76** | **77** | **78** | **79** | **80** | **81** | **82** | **83** | **84** |
| WHITE LIGHT SET #2 (WL 2) | **85** | **86** | **87** | **88** | **89** | **90** | **91** | **92** | **93** | **94** | **95** | **96** |
| FLUORESCENCE (FL) | **97** | **98** | **99** | **100** | **101** | **102** | **103** | **104** | **105** | **106** | **107** | **108** |
